# Detection of key mRNAs in liver tissue of hepatocellular carcinoma patients based on machine learning and bioinformatics analysis

**DOI:** 10.1016/j.mex.2023.102021

**Published:** 2023-01-18

**Authors:** Maryam Gholizadeh, Seyed Reza Mazlooman, Morteza Hadizadeh, Marek Drozdzik, Saeid Eslami

**Affiliations:** a Department of Medical Informatics, Faculty of Medicine, Mashhad University of Medical Sciences, Mashhad 91388-13944, Iran; b Department of Computer Engineering, Central Tehran Branch, Islamic Azad University, Tehran 1477893780, Iran; c Physiology Research Center, Institute of Neuropharmacology, Kerman University of Medical Sciences, Kerman 7616913555, Iran; d Department of Experimental and Clinical Pharmacology, Pomeranian Medical University, Szczecin 70-111, Poland

**Keywords:** Non-fusion integrative method, Meta-analysis, Machine learning, Survival analysis, Non-fusion integrative Meta-analysis

## Abstract

One methodology extensively used to develop biomarkers is the precise detection of highly responsive genes that can distinguish cancer samples from healthy samples. The purpose of this study was to screen for potential hepatocellular carcinoma (HCC) biomarkers based on non-fusion integrative multi-platform meta-analysis method. The gene expression profiles of liver tissue samples from two microarray platforms were initially analyzed using a meta-analysis based on an empirical Bayesian method to robust discover differentially expressed genes in HCC and non-tumor tissues. Then, using the bioinformatics technique of weighted correlation network analysis, the highly associated prioritized Differentially Expressed Genes (DEGs) were clustered. Co-expression network and topological analysis were utilized to identify sub-clusters and confirm candidate genes. Next, a diagnostic model was developed and validated using a machine learning algorithm. To construct a prognostic model, the Cox proportional hazard regression analysis was applied and validated.

We identified three genes as specific biomarkers for the diagnosis of HCC based on accuracy and feasibility. The diagnostic model's area under the curve was 0.931 with confidence interval of 0.923–0.952.

• Non-fusion integrative multi-platform meta-analysis method.
• Classification methods and biomarkers recognition via machine learning method.
• Biomarker validation models.

## Graphical abstract

**Figure:**
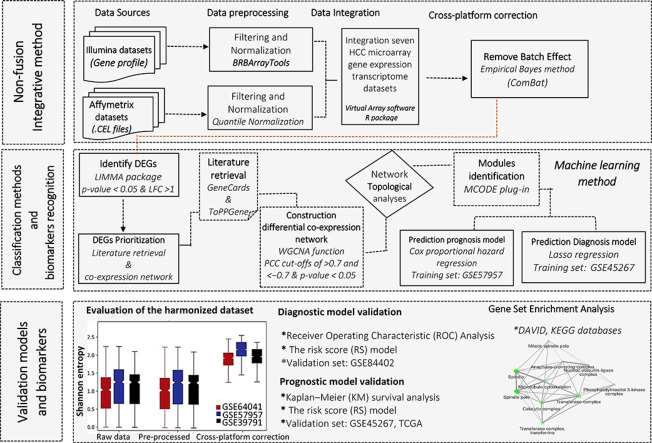

Specifications table

**Table:** 

Subject area:	Bioinformatics
More specific subject area:	Computational genomics, system biology and biomarker detection
Name of your method:	Non-fusion integrative Meta-analysis
Name and reference of original method:	**Name:** Integrative multi-platform meta-analysis **Reference:** doi 10.1016/j.gendis.2022.07.018
Resource availability:	1.GEO database (https://www.ncbi.nlm.nih.gov/geo/) 2. TCGA database (https://cancergenome.nih.gov/) 3. GeneCards (https://genecards.org) 4. ToPPGene (https://toppgene.cchmc.org) 5. Cytoscape (http://apps.cytoscape.org/apps/mcode) 6. KM plotter (http://www.kmplot.com/analysis/) 7. DAVID database (https://david.ncifcrf.gov/) 8. GO database (http://geneontology.org/) 9. KEGG database (https://www.genome.jp/kegg/pathway.html)

## Background information

Identifying key genes that can distinguish cancers from normal samples is one of strategy widely utilized to develop diagnostic biomarkers. Several research aimed to identify diagnostic and prognostic biomarkers by splitting the most informative genes from the irrelevant ones. Dessie et al. used statistical and bioinformatics tools to identify HCC biomarkers based on Differentially Expressed Genes (DEGs) [1]. For the diagnosis of liver hepatocellular cancer, Ouyang *et al*. provided 34 DEGs based on several machine learning techniques [2]. Microarray data are now widely used to address biological questions. This has resulted in an explosion of raw microarray data from various chip platforms. As a result, databases with hundreds of thousands of microarray samples clustered by different chips are difficult to merge. One of the primary goals of these databases was to make the data available to other researchers for more accurate analysis with large sample size. Therefore, using a meta-analysis to integrate data from various platforms is critical, as it can significantly improve the reliability and robustness of biomarker detection. Most of earlier investigations used only data sets generated on the same chip platform. So, the prediction accuracy and application scope of these studies have been severely limited by sample size.

In this study, we aimed to identify and validate diagnosis and prognosis biomarkers associated with HCC based on expression data of two microarray chip platforms. Based on a meta-analysis, 939 samples (493 tumors and 446 non-tumors) from the Gene Expression Omnibus (GEO) were screened to identify DEGs. Weighed correlation network analysis (WGCNA) method was used to cluster the prioritized DEGs. The co-expression networks and topological analysis were performed to find the sub-clusters and confirm target genes. Finally, based on these genes two prediction models, involved in the diagnosis and prognosis, were established. The gene expression profile of GSE45267 was applied as a training cohort to build a Lasso regression model for diagnosis and GSE84402 was used as a validation cohort. In addition, to verify the prognostic ability of the risk score model, GSE57957 gene expression data was utilized as a training cohort, and GSE45267 and TCGA (https://cancergenome.nih.gov/) data were used as validation cohorts.

## Method details

### Non-fusion integrative method

#### HCC Illumina and Affymetrix datasets

In this investigation, which was based on the Illumina and Affymetrix platforms, seven microarray gene expression transcriptome datasets were considered. Human samples were divided into the HCC group and adjacent or non-tumor groups; sample numbers greater than ten for the HCC and non-tumor groups were included in each dataset; and only mRNA expression profiling was employed to the study. Additionally, the included dataset met the criteria for demographic characteristics, etiology, and Edmonson stage (Table 1). Based on the TCGA dataset, which contains 371 HCC and 50 normal tissue samples, the important HCC prognostic genes were discovered.

**Table 1:** Dataset with the following baseline characteristics were considered.

Datasets	Sex: Male)%)	Age mean ± SD	Etiology	Stages (Edmonson)	Platform	Application
GSE57957	90	65±20	HBV infection	I, II, III, IV	Illumina	DEGs Identification, prognostic model identification
GSE39791	84	57.5 ± 20	HBV infection	I, II, III, IV	Illumina	DEGs Identification
GSE36376	83	55.9 ± 10	HBV and HCV	I, II, III, IV	Illumina	DEGs Identification
GSE84005	86	54.5 ± 18	HBV and HCV	I, II, III	Affymetrix	DEGs Identification
GSE12941	100	65.5 ± 10	HBV and HCV	I, II, III	Affymetrix	DEGs Identification
GSE64041	88	64 ± 12	HBV and HCV	I, II, III, IV	Affymetrix	DEGs Identification
GSE45267	90	51 ± 14	HBV and HCV	I, II, III, IV	Affymetrix	Diagnostic model and prognostic model validation
GSE84402	30	55 ± 11	HBV and HCV	I, II, III, IV	Affymetrix	DEGs Identification and diagnostic model validation

### Cross-platform correction

The data was preprocessed and integrated using the Irigoyen et al. method [3]. Each experiment's data was imported, filtered, and normalized independently. For preprocessing data from Affymetrix platforms, we used BRBArrayTools, an Excel GUI for interacting with the R programming environment. The R package Lumi was used to perform quantile normalization to Illumina expression data. To reduce false-positive rates, we removed genes with minimal expression variability (p-value < 0.05). Transcriptome datasets were integrated using the virtual array software R program. This software enables the integrate data from several microarray chip using batch effect and cross-platform correction. Empirical bayes approach (ComBat) was used to eliminate heterogeneity [4].

## Classification methods and biomarkers recognition

### Analysis of differential gene expression patterns

Linear models for microarray data (LIMMA) software R package based on the Bayesian technique was used to identify DEGs from normalized log-expression levels in the integrated data. The false discovery rate was managed using Benjamini-Hochberg's method. A p-value threshold of 0.05 was ad-judged statistically significant with a fold-change cutoff of 1 (Log FC >1).

### DEGs elimination based on literature retrieval and co-expression network

#### Literature retrieval

GeneCards and ToPPGene websites were used to identify and prioritize DEGs with high confidence. The literary evidence for reported genes was extracted from the GeneCards website (training group). The used keywords included “hepatocellular carcinoma” + “biomarker” and “hepatocellular carcinoma” +” DEGs”. Then, the ToPPGene website was used to order the test group of genes based on the training group to discover the most significant DEGs in HCC patients with a p-value less than 0.05.

#### Co-expression network

A differential co-expression network among DEGs was developed in accordance with the WGCNA method, in order to group genes with strong correlation and discover co-expression modules. The STRING database was used to develop a protein-protein interaction network with the highest confidence threshold in order to better understand the interconnectivity of DEGs (0.9). A matrix of pairwise Pearson's correlation coefficients was used to evaluate the level of correlation between the gene expression profiles. With the WGCNA function adjacency, we were able to create an adjacency matrix from the similarity matrices computed using Pearson correlation coefficients (PCCs). The pairwise correlations' statistical significance was assessed using the PCC cut-offs of 0.7, which correspond to p-value < 0.05. Cytoscape (v.3.7.1) was used to illustrate the final outcome which was done using the Cytoscape plug-in Molecular Complex Detection with the following parameters: degree cut-off = 2, node score cut-off = 0:2, kcore = 2, and maximum depth = 100 and CytoHuba. We examined modules with at least ten nodes in deeper detail. Clusters of differentially expressed genes were discovered and visualized by using the MCODE plug in. Diagnostic and prognostic information was derived from genes found in sub-clusters [5].

### Local and global network metrics analyses

With plug-in Network-Analyzer of Cytoscape [6], we performed topological analysis to the network in order to identify the hub genes, which included metrics including degree, betweenness connectivity, network density, and clustering coefficient. In terms of degree and betweenness, the top five molecule candidates were identified as hubs.

### Diagnostic risk model

In order to identify diagnostic gene biomarkers, the least absolute shrinkage and selection operator (lasso) regression model was developed. Many regression analysis methods utilize the Lasso regression regularization. The goal of lasso is to reduce the weighted average of mean squared prediction error for samples by identifying regression coefficients for genes [1]. In order to build a diagnostic model, we used the gene expression profile of GSE45267. Consequently, the risk score model for Lasso regression was built using the LARS package [7].

### Prognostic risk model and survival analysis

To develop a prognostic model, the gene expression profile of GSE57957 was used. Then a prognostic model was constructed based on a linear combination of the regression coefficient derived from the Lasso Cox regression model coefficients multiplied with its mRNA expression level. The statistical significances of OS to compare the survival difference between the high- and low-expression groups, were determined using the Log-Rank test. The median was chosen as a cutoff value to make dividing line between high-risk and low-risk patients and OS with the best performing threshold. The results were displayed with criteria of hazard ratios (HRs) > 1 or HRs < 1. The "survminer" package was used to visualize the survival curves. The survival probability at any particular time was calculated by the number of subjects surviving (the number of living- the number of died) divided by the number of subjects living at the start.

## Validation models and biomarkers

**Fig. 1:**
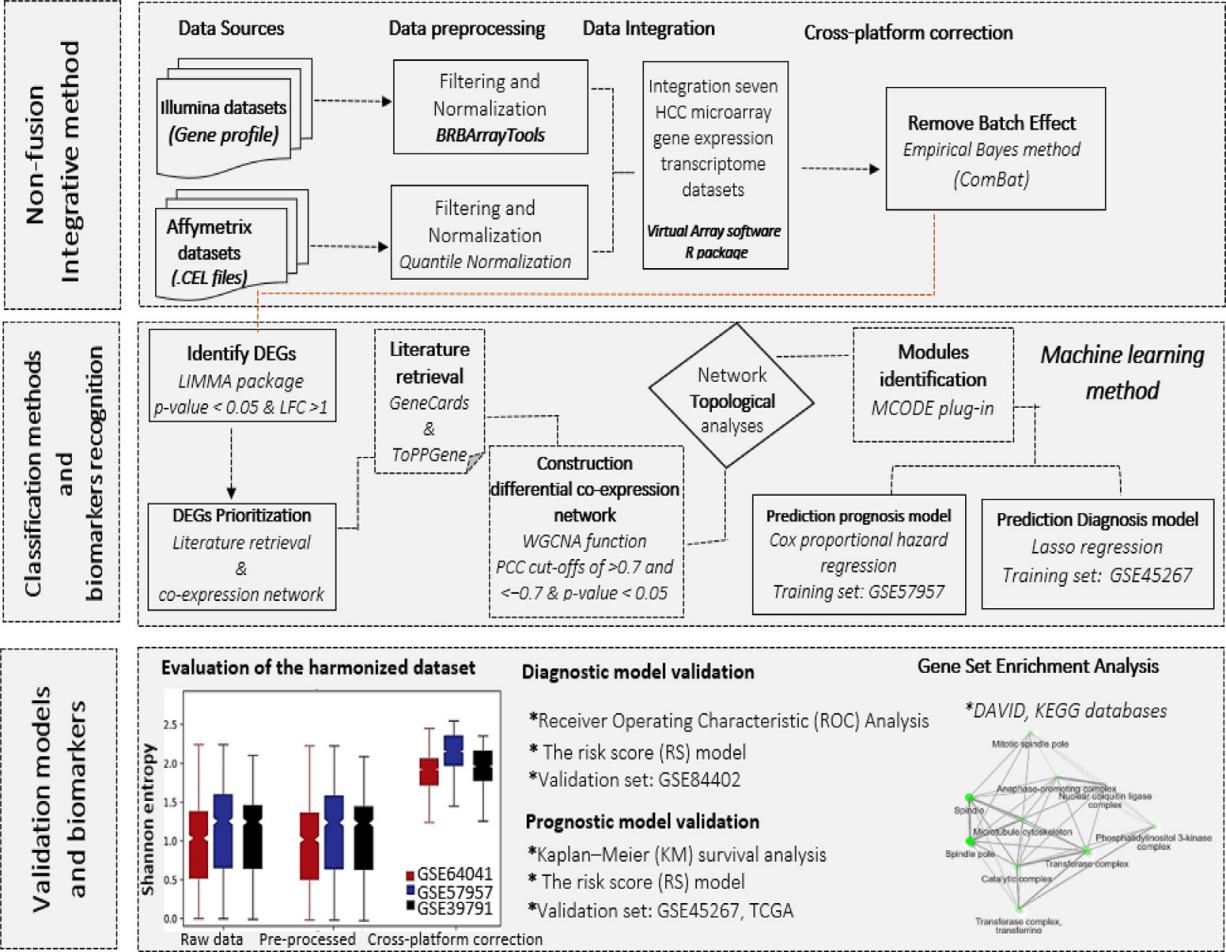
Three-step identification of HCC potential biomarkers. First, DEGs using non-fusion integrative method detected. Based on ComBat approach, the batch effect between data from two main microarray platforms (Affymetrix and Illumina), removed. Second, to deal with a more in-depth analysis of HCC expression datasets to identify potential diagnosis and prognosis biomarkers, classification methods and biomarker recognition conducted. Third, to evaluate the diagnostic performance in classifying HCC from normal, the risk score model was developed, and to assess the prognostic value of gene biomarkers, Kaplan–Meier (KM) survival analysis performed.

**Fig. 2:**
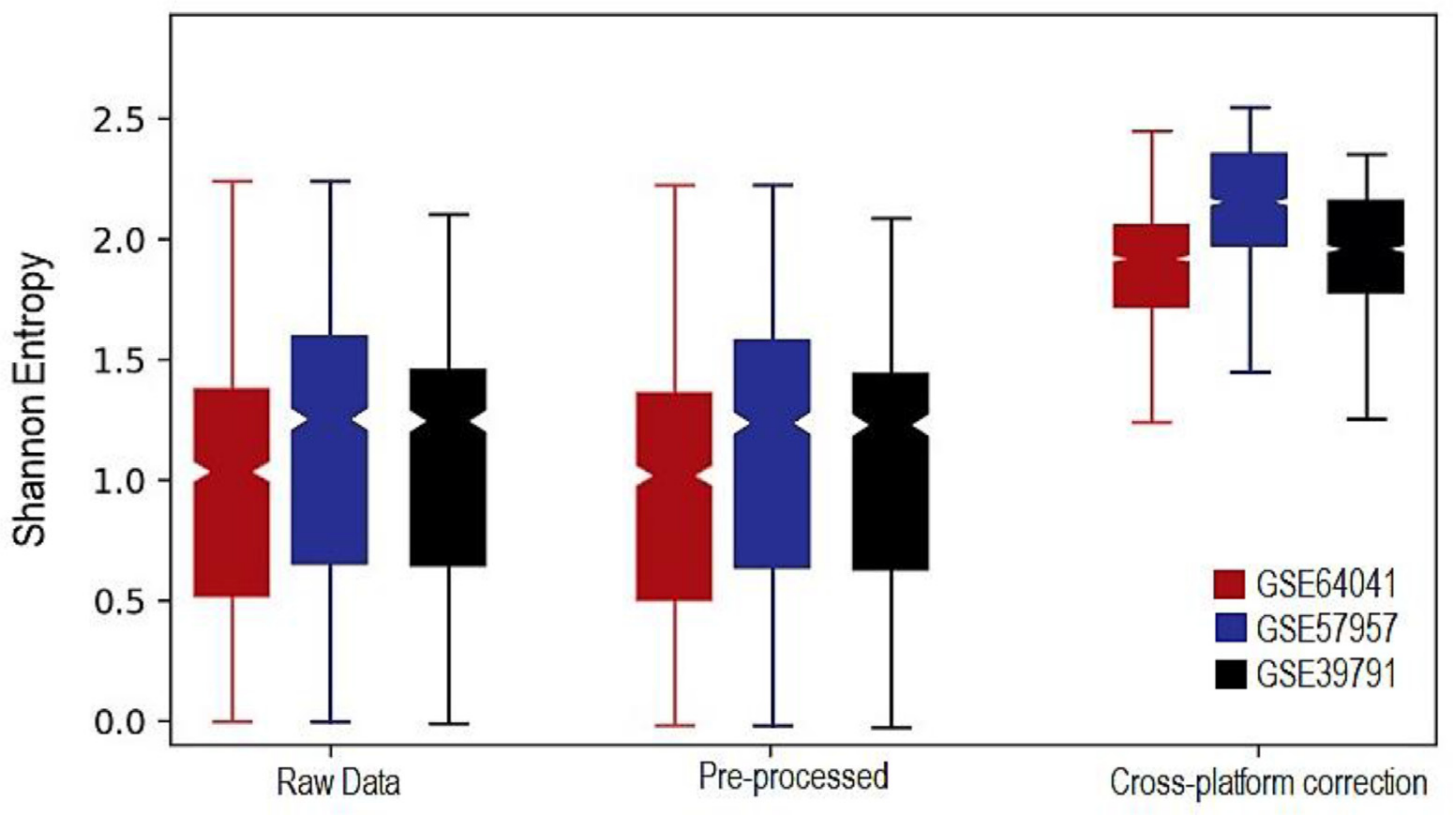
Performance assessment from our remove heterogeneity method. In order to quantify the correction success, we calculated the local Shannon entropy. Box plot is confirmed how data were integrated and heterogeneity removed.

It is challenging to accurately measure whether batch effect correction method is working especially without a reference data set. Visual evaluation is a common method for determining the efficacy of a batch adjustment. Following, we calculated the local Shannon entropy, which each sample k the entropy S based on batch b is defined as;

$$S_{k}=-\sum_{b=1}^{n_{b}}p_{b}\ln\left(p_{b}\right)$$

where p_(b)is the estimated probabilities of the different batches and n_b is the total number of batches. According to the comparative boxplot, the ComBat was shown to be beneficial in this research (Figs. 1 and 2). Diagnostic Risk score model's superiority was evaluated using receiver operating characteristic (ROC) analysis. Based on ROC analysis of the training set (AUC = 0.952) the co-detection of these genes displayed a great performance of the model in HCC diagnosing. The gene expression profile of GSE84402 was used as a validation set. Diagnostic performance in distinguishing between HCC and normal was evaluated using the risk score model. The AUC in the validation set was 0.941 (*P*<0.0001). The prognostic ability of a risk score model was then addressed using expression data from GSE45267 and TCGA as a validation set. Gene biomarkers were evaluated using Kaplan–Meier (KM) survival analysis. An analysis of the relationship between overall survival (OS) time and gene biomarker obtained by lasso was performed using the KM plotter tool. Genome ontology (GO) and Kyoto Encyclopedia of Genes and Genomes (KEGG) were performed for the representative genes to assess the potential functional role and biological mechanism behind them. The gene ontology related to different subtypes was found using the database for annotation, visualization, and integrated discovery (DAVID) database. The R code of this study is included as supplementary file *Non-fusion_integ_microdata.R*.

## Additional information

To improve the reliability and robustness of possible biomarker detection to enhance decision-making for HCC patient management, we performed an integrated meta-analysis of various MAGE datasets. To eliminate confounding influences in the data analysis, Affymetrix GeneChips and Illumina BeadChips data were preprocessed separately. The ComBat technique was used for batch correction and data integration. There is a vast amount of literature, which strongly recommends ComBat due to its (i) low computational cost (ii) independence of sample size (iii) reduced inter-platform variance (iv) outperformance of other approaches such as DWD or MC.

**Fig. 3:**
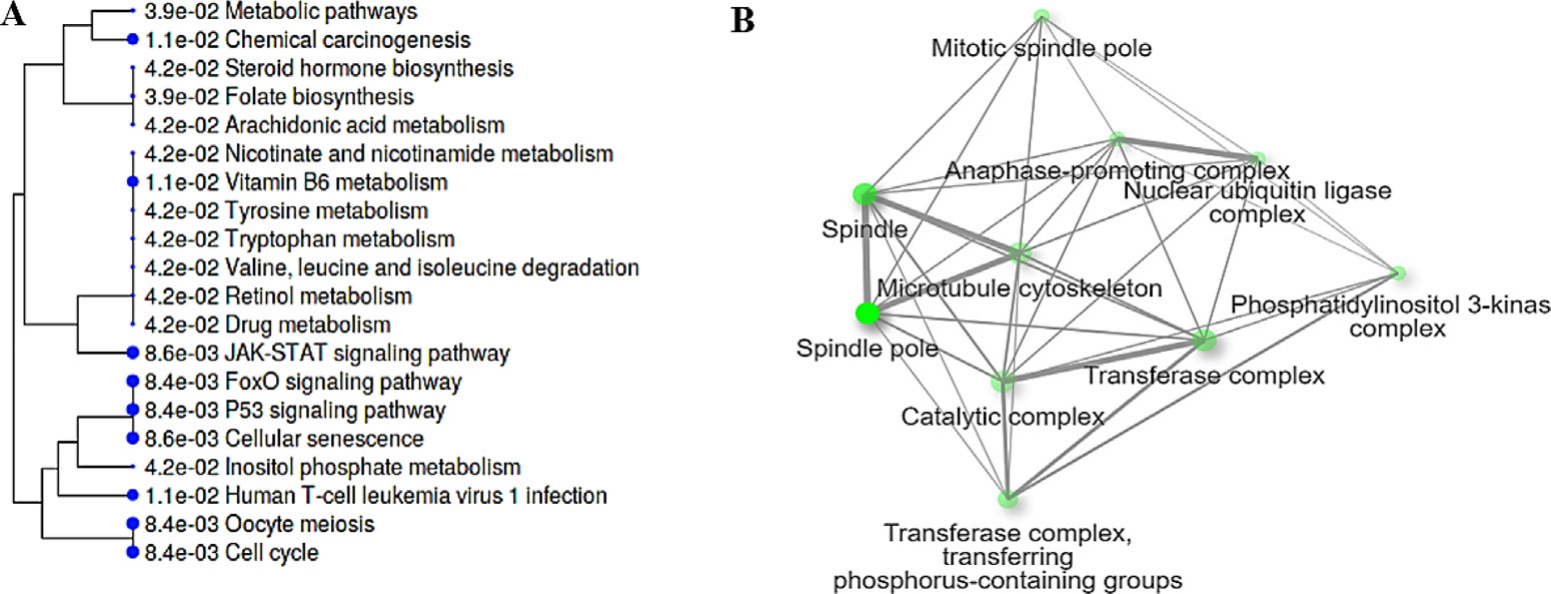
Cellular component (A) and Pathway and (B) of SOCS2, AOX1, CCNB1, PTEN, FAM83D, AKR1C3, CDC20; 7 DEGs were identified as being common in all three analyzes- Affymetrix, Illumina, and integrated meta-analysis.

After integration, 302 DEGs were identified (LFC > 1 and FDR < 0.05), and 7 DEGs were identified as being common in all three analyzes- Affymetrix, Illumina, and integrated meta-analysis. These genes, namely SOCS2, AOX1, CCNB1, PTEN, FAM83D, AKR1C3, CDC20 were shown to be potential biomarkers for HCC given in prior research. However, despite remarkable biomedical researches, there is still an urgent need to find disease-specific and effective molecular signatures, since investigations have focused on single genes associated with HCC, ignoring the interactions and associations among them. The construction of DCENs from DEGs using pairwise correlation metrics and their topological analysis gives critical insights and enlightens us about the changes that occur in biological systems as a result of environmental and biological disturbances caused by disease. In several studies, human disease-associated genes and gene clusters of highly connected network components were identified through weighted correlation network analysis, including chronic lymphocytic leukemia [8], obesity [9] tumor-associated macrophages [10], breast cancer [11,12], ovarian cancer [13], hepatocellular carcinoma, and cholangiocarcinoma [14], pancreatic cancer [15], cholangiocarcinoma [16], lung cancer [17] and HCC [18]. Li et al. built an integrated co-expression network via three gene expression profiles of 480 patients with HCC to investigate the hub genes and biological processes of HCC, which change substantially during its progression [18]. In the present study, 239 prioritized DEGs represented three DCEMs. The cooperative role among modules might enhanced as a result of the modification and may have a strong correlation with tumor evolution. Interestingly, the majority of those differential co-expressed genes coded for proteins taking roles in the Wnt–β-catenin signaling pathway, NF-κB pathway, cell cycle, and p53 pathway (Fig. 3).

Studies have reported, in around 40% of the cases, significantly higher activation of the Wnt–β- catenin signaling pathway, caused by genetic alterations in CTNNB1, TP53, RB1, CCNA2, CCNE1, PTEN, ARID1A, ARID2, RPS6KA3 or NFE2L2, all of which are involved in cell cycle control [19,20]. Several examinations have also highlighted the NF-κB pathway as a key inflammatory signaling pathway involved in HCC induction [21,22]. The topological analysis resulted in the identification of five top hub genes with three repressed (CCNB1, IGF1, SOCS2) and two overexpressed (AFP, CDK4). However, functional enrichment analyses of these hub genes showed the predicted results; the majority of significantly enriched genes were involved in metabolic and cellular processes. CDK4, which is involved in the regulation of cell proliferation, apoptosis, and drug resistance, is also being investigated as a target for HCC chemotherapeutics in preclinical and clinical trials. Notably, many studies reported that overexpression of CDK4 mRNA and protein promoted HCC progression and poor prognosis. Moreover, CDK4 and its direct correlation with clinical parameters, tumor stage, size, and poor survival rate in HCC patients have been revealed [23], [24], [25], [26].

Finally, several genes have been proven to be the prognostic or diagnostic biomarkers of HCC. Clinical symptoms, pathological classification, and gene expression data could be used to build cancer prognostic and diagnostic predictive models. Early detection of cancer has always been challenging. In the present study, a set of nine robust diagnostic signatures were identified by Lasso regression analysis, and then, a three-gene simplified diagnostic risk model compositing CYP2E1, AKR1C3, AFP was built. Among these genes, AFP as an extensively researched biomarker for HCC has been reported by several studies [27], [28], [29], [30], [31], as the diagnostic biomarker in HCC patients. Nonetheless, recently reported the use of AFP as a biomarker for early diagnosis of HCC has been limited because of the high false-positive rate and its low sensitivity (55%). Furthermore, its overexpression is detected in other liver abnormalities and other tumors, consequently, the specificity of this biomarker is not too high (87%) [30]. Nevertheless, the isoform of AFP namely AFP-L3 is suggested to be a remarkably more reliable biomarker because of expression increase only in HCC but not in hepatitis or cirrhosis cases. CYP2E1 is an enzyme important in ethanol metabolism, and its increased activity was documented in HCC using chlorzoxazone as a probe drug [32]. CYP2E1 was also documented as one of the DEGs as analyzed in the GSE36376 dataset using GEO2R [33]. Those observations are in keeping with HCC clinical features, i.e. the 5-year cumulative HCC incidence in cirrhosis was estimated to be 8% in alcoholic cirrhosis [34]. AKR1C3 (Aldo-keto reductase family 1 member C3) may have a role in controlling cell growth and/or differentiation. Likewise, to the present study, Zhu et al. applying bioinformatics methods suggested AKR1C3 overexpression in HCC and identified its diagnostic and prognostic value [35]. The study also addressed the possible underlying molecular mechanisms, showing that AKR1C3 might participate in the MAPK/ERK and androgen receptor signaling pathways.

Moreover, in the present study, a four-gene prognostic signature including SOCS2, MAGEA6, RDH16, and RTN3 was established by Cox proportional hazards regression model combined with Kaplan-Meier survival analysis and could predict the overall survival of HCC. We demonstrated that SOCS2, RDH16 expression was significantly downregulated in HCC, and MAGEA6, RTN3 were significantly upregulated as compared with normal liver tissues. SOCS2 is a member of the SOCS family and several studies have been reported its close association with HCC and its role in the inhibition of tumor metastasis [36], [37], [38]. Hence, SOCS2 may provide a useful HCC treatment and diagnostic target. Liu et al., *via in vitro* and *in vivo* experiments demonstrated that overexpression of SOCS2 inhibited HCC cell proliferation and migration, whereas SOCS2 knock‑down promoted HCC tumorigenesis suggesting that SOCS2 may act as a potential HCC prognostic biomarker [38]. RDH16 (coding for retinol dehydrogenase 16) is another downregulated gene in the present study. This observation is in keeping with other repost [39], which also demonstrated RDH16 protein suppression. Functional experiments showed that ectopic expression of RDH16 in HCC cells suppressed cell growth, clonogenicity, and cell motility, and was associated with increased levels of retinoic acid, which was widely evidenced to inhibit tumor development and progression [40]. A stratified survival analysis based on the clinical stage demonstrated that higher expression of RDH16 predicted better prognosis of HCC patients in early clinical stage. The present study also revealed up-regulation of MAGEA6 and RTN3 expression as prognostic features in HCC. Recent studies are in keeping with our findings, and revealed MAGEA6 (melanoma antigen A6) among up-regulated genes in HCC, also evidencing its prognostic value [41,42]. MAGEA6 functions as a ubiquitin ligase physiologically, and normally is expressed only in the male germline, but also can be re-activated in human cancers. In neoplastic cells, MAGEA6 and TRIM28 are combined to form a cancer-specific ubiquitin ligase, that leads to inhibition of the AMPK signaling pathway, and in HCC induced the stemness maintenance and selfrenewal of stem cells [43]. The upregulation of the expression of reticulon 3 (RTN3) is a component of the prognostic signature revealed in the present study. This finding is supported by observations of others, who also classified RTN3 increased expression as positive prognostic marker [44]. Recently is has been evidenced that RTN3 restrained HCC growth and induced apoptosis. These effects were mediated by p53 activation of p53, i.e. RTN3 facilitated p53 Ser392 phosphorylation via Chk2 (RTN3 recruited Chk2 to the endoplasmic reticulum and promoted its activation) and enhanced subsequent p53 nuclear localization. RTN3 interacted with Chk2 [44].

So, the reported in the present study the three-gene diagnostic signature CYP2E1, AKR1C3, AFP as well as the four-gene prognostic signature including SOCS2, MAGEA6, RDH16, and RTN3 are supported by the potential biological role of the respective proteins in the pathogenesis of HCC.

## Conclusions

To improve the reliability and robustness of potential biomarkers detection, an innovative analysis has been performed with the large accumulation of seven HCC transcriptome datasets. Our results show despite, the abundance of transcriptomic data provided by different platforms, non-fusion integrative meta-analysis could be a compelling method when aiming for detection diagnostic and prognostic biomarkers.

## Related research article

M. Gholizadeh, M. Hadizadeh, S.R. Mazlooman, S. Eslami, S. Raoufi, M. Farsimadan, M. Rashidifar, M. Drozdzik, M. Mehrabani. Integrative multi-platform meta-analysis of hepatocellular carcinoma gene expression profiles for identifying prognostic and diagnostic biomarkers. Journal of Genes and Diseases, In press. doi 10.1016/j.gendis.2022.07.018

## CRediT authorship contribution statement

**Maryam Gholizadeh:** Conceptualization, Methodology, Software, Validation, Writing – review & editing. **Seyed Reza Mazlooman:** Methodology, Validation, Data curation, Writing – original draft. **Morteza Hadizadeh:** Visualization, Investigation, Writing – review & editing, Writing – original draft. **Marek Drozdzik:** Supervision, Conceptualization, Writing – review & editing. **Saeid Eslami:** Supervision, Conceptualization, Writing – review & editing, Writing – original draft.

## Declaration of Competing Interest

The authors declare that they have no known competing financial interests or personal relationships that could have appeared to influence the work reported in this paper.

## Data Availability

Data will be made available on request.
